# Optimized Bone Regeneration in Calvarial Bone Defect Based on Biodegradation-Tailoring Dual-shell Biphasic Bioactive Ceramic Microspheres

**DOI:** 10.1038/s41598-018-21778-z

**Published:** 2018-02-21

**Authors:** Antian Xu, Chen Zhuang, Shuxin Xu, Fuming He, Lijun Xie, Xianyan Yang, Zhongru Gou

**Affiliations:** 1 The Affiliated Stomatology Hospital, School of Medicine of Zhejiang University, Hangzhou, 310006 China; 2 Bio-nanomaterials and Regenerative Medicine Research Division, Zhejiang-California International Nanosystem Institute, Zhejiang University, Hangzhou, 310058 China; 3 Department of Orthopaedic Surgery, the Second Affiliated hospital, School of Medicine of Zhejiang University, Hangzhou, 310009 China

## Abstract

Bioceramic particulates capable of filling bone defects have gained considerable interest over the last decade. Herein, dual-shell bioceramic microspheres (CaP@CaSi@CaP, CaSi@CaP@CaSi) with adjustable beta-tricalcium phosphate (CaP) and beta-calcium silicate (CaSi) distribution were fabricated using a co-concentric capillary system enabling bone repair via a tailorable biodegradation process. The *in vitro* results showed the optimal concentration (1/16 of 200 mg/ml) of extracts of dual-shell microspheres could promote bone marrow mesenchymal cell (BMSC) proliferation and enhance the level of ALP activity and Alizarin Red staining. The *in vivo* bone repair and microsphere biodegradation in calvarial bone defects were compared using micro-computed tomography and histological evaluations. The results indicated the pure CaP microspheres were minimally resorbed at 18 weeks post-operatively and new bone tissue was limited; however, the dual-shell microspheres were appreciably biodegraded with time in accordance with the priority from CaSi to CaP in specific layers. The CaSi@CaP@CaSi group showed a significantly higher ability to promote bone regeneration than the CaP@CaSi@CaP group. This study indicates that the biphasic microspheres with adjustable composition distribution are promising for tailoring material degradation and bone regeneration rate, and such versatile design strategy is thought to fabricate various advanced biomaterials with tailorable biological performances for bone reconstruction.

## Introduction

Bone substitute materials are frequently required to repair bone defects due to any trauma, disease and surgery. It is generally agreed that the synthetic bioceramics and composites, are virtually unlimited available for bone replacement^1^. As an important representative, hydroxyapatite (HA) bioceramic is available clinically in orthopedics and dentistry because of its similar chemical composition to bone mineral and the ability to promote osteointegration^2^. However, the HA bioceramic is sparingly bio-degradable and lack the ability to stimulate osteogenic cell differentiation and bone regeneration, which impede their wider clinical application^3,4^.

On the other hand, the beta-tricalcium phosphate (Ca_3_(PO_4_)_2_; β-TCP) and beta-calcium silicate (CaSiO_3_; β-CaSi) have both received great interests for respective special characteristics and thus have been extensively investigated as bone substitutes^5–7^. β-TCP has been used clinically as bone substitute due to its good osteoconduction^5,6^. However, its degradation rate tends to be slow, not satisfying the requirement of expected bone regeneration in bone defects^7^. Also, it is generally considered that β-TCP lacks osteostimulatory ability, which would affect its clinical applications^8,9^. In contrast, the presence of silicon in β-CaSi ceramics confers its osteostimulatory feature, which make this bioceramic promising bone substitutes^10^. Some studies have demonstrated that β-CaSi ceramic soaked in various conditions such as simulated body fluid (SBF), human saliva, and cell culture medium could induce apatite mineralization^11–13^. Silicon ions released from such Ca-silicate ceramic can stimulate osteogenesis and angiogenesis by promoting osteogenic differentiation of bone marrow derived stroma cells and improving human umbilical vein endothelial cells angiogenesis^14–16^. Xu *et al*. observed that β-CaSi porous scaffolds could stimulate appreciable new bone tissue ingrowth in the early stage in comparison with β-TCP scaffolds^7^. However, a major drawback of β-CaSi porous materials is their too fast biodegradation that may sacrifice structural and mechanical support for the newly formed bone remodeling^17^. Therefore, an ideal bone scaffold should be highly bioactive (osteoconductive & osteostimulative) and meanwhile, could be resorbed gradually in step with new bone regeneration and repair.

Nowadays, it is well agreed that a combination of β-TCP and β-CaSi may have synergistic effects, which fill the respective gap between material resorption and bone regeneration, ultimately facilitating the bone regeneration and remodeling^17,18^. There are some studies of porous scaffolds via mechanically mixing β-TCP/β-CaSi with different β-TCP mass fraction^17,19–21^ but little of research on excellent osteogenic capability has been confirmed. On the other hand, granular form of bioceramics has an advantage of suiting for non-geometrically well-defined craniomaxillofacial bone defects accompanied with favorable interconnected macropore structures and is also potentially used as drug delivery systems^22,23^. Recently, we have developed a gradient hybrid design to fabricate core-shell β-TCP‒β-CaSi (CaP@CaSi, CaSi@CaP) microspheres conceiving respective biological performance to be exerted in specific layer^24^. Our studies have demonstrated that the concentrations of silicon (Si), calcium (Ca), and phosphate (P) ions in Tris buffer released from such layered microspheres were closely related with the composition distribution of bioceramics, and thus the degradation rate of each ceramic phase could be modulated through its concrete position in the core-shell architectures. More recently, another study has confirmed the CaP shell layer of CaSi@CaP microspheres was particularly beneficial for bone marrow mesenchymal stem cell (BMSC) adhesion and growth, and the ion release from the CaP@CaSi exhibited a potent stimulating effect on alkaline phosphatase (ALP) expression of cells at 10 days after culture; meanwhile, the pure CaSi and CaP ceramic microspheres were both unfavorable for osteogenesis due to too fast or slow biodegradation *in vivo*^18^. The results from these two studies emphasized the fact that changes in the composition distribution have significant effects in the time-dependent physicochemical properties of the CaSi‒CaP biphasic composites as well as the biological performances of final products.

Based on the aforementioned perspective, the present study aimed to examine whether the gradient tri-layer distribution of slowly degraded β-TCP (osteoconductive phase) and quickly degraded β-CaSi (osteostimulative phase) could enhance bone regeneration in thin-wall bone defects. Two groups of bioceramic granules were prepared including β-TCP or β-CaSi centered tri-layer microspheres (CaP@CaSi@CaP, CaSi@CaP@CaSi) by being distributed in separate core or shell layers. The cell responses of these biomaterials with different biodegradation characteristics were studied *in vitro*. Meanwhile, these microspheres were evaluated in thin-wall calvarial defect models in rabbits, in comparison with the pure β-TCP granules (i.e. CaP microspheres). The osteogenesis and material degradation efficiency was systematically evaluated by micro-computed tomography technology (μCT) at 6‒18 weeks postoperatively, and the sequential histological evaluations and bone regeneration mechanism were unveiled as well.

## Results

### Characterization of bioceramic microspheres

**Figure 1:**
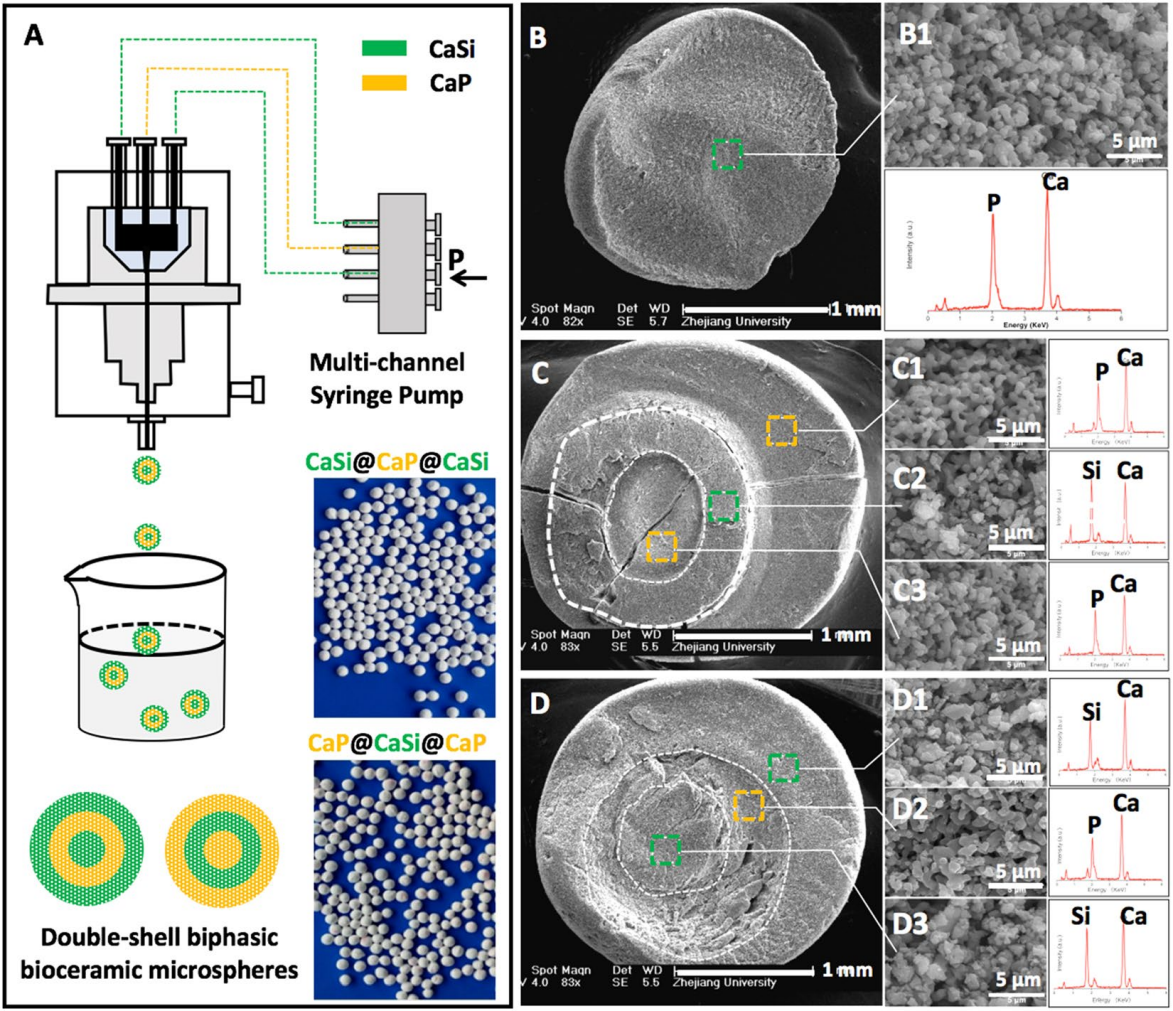
Schematic illustration of preparation of double-shell biphasic bioceramic microspheres (**A**) and SEM observation of the fracture surface of (**B**) CaP, (**C**) CaP@CaSi@CaP, and (**D**) CaSi@CaP@CaSi microspheres. The dotted-frames representing the selected-area high magnification of SEM images (B1, C1–3, D1–3) and the face-scanning EDX spectra aside showing the chemical component of corresponding layer. The light image (**A**) showing the as-sintered representative microspheres. The dot-line (**B**,**C**,**D**) showing the interface of the different layers.

Figure 1A illustrates that the bioceramic microspheres formed through tri-nozzle systems and the schematic illustration of the core-shell structure of the microspheres. The optical images revealed the monodisperse microsphere morphology. SEM images (Fig. 1B‒D) indicated the dual-shell feature in the CaP@CaSi@CaP and CaSi@CaP@CaSi, and the area-selected EDX spectra also confirmed the corresponding chemical composition in different layers, which was consistent with the CaP or CaSi component in the ceramic slurries during the preparation of the microspheres. Moreover, the high-magnification SEM images showed that the as-sintered core or shell component in the microspheres exhibited low-densification porous structures.

### Ionic concentrations of extracts

The Ca, P, and Si concentrations in the microsphere extracts were shown in Table 1. The CaP released appreciable amount of P ions (18.42 ± 2.76 ppm) into the cell culture medium in comparison with that (4.70 ± 0.31 ppm) in the primary DMEM. The CaSi@CaP@CaSi extract had significantly high Ca concentration (38.06 ± 3.54 ppm), which was nearly 2-fold than that in the CaP@CaSi@CaP extract (*p* < 0.05). In particular, it is worth noting that the dual-shell microsphere extracts had a comparatively high Si concentration (>100 ppm), while the Si concentration in the pure CaP extract were slightly detected (<2 ppm).

**Table 1:** The ionic concentrations of extracts.

Group	Ca (ppm)	P (ppm)	Si (ppm)
DMEM	10.83 ± 0.25	4.70 ± 0.31	0.16 ± 0.04
CaP	13.93 ± 1.27	18.42 ± 2.76	1.36 ± 0.85
CaP@CaSi@CaP	19.93 ± 1.61	6.89 ± 0.44	109.86 ± 6.82
CaSi@CaP@CaSi	38.06 ± 4.54	11.28 ± 0.39	126.69 ± 5.79

### Proliferation of BMSCs cultured in conditioned media

**Figure 2:**
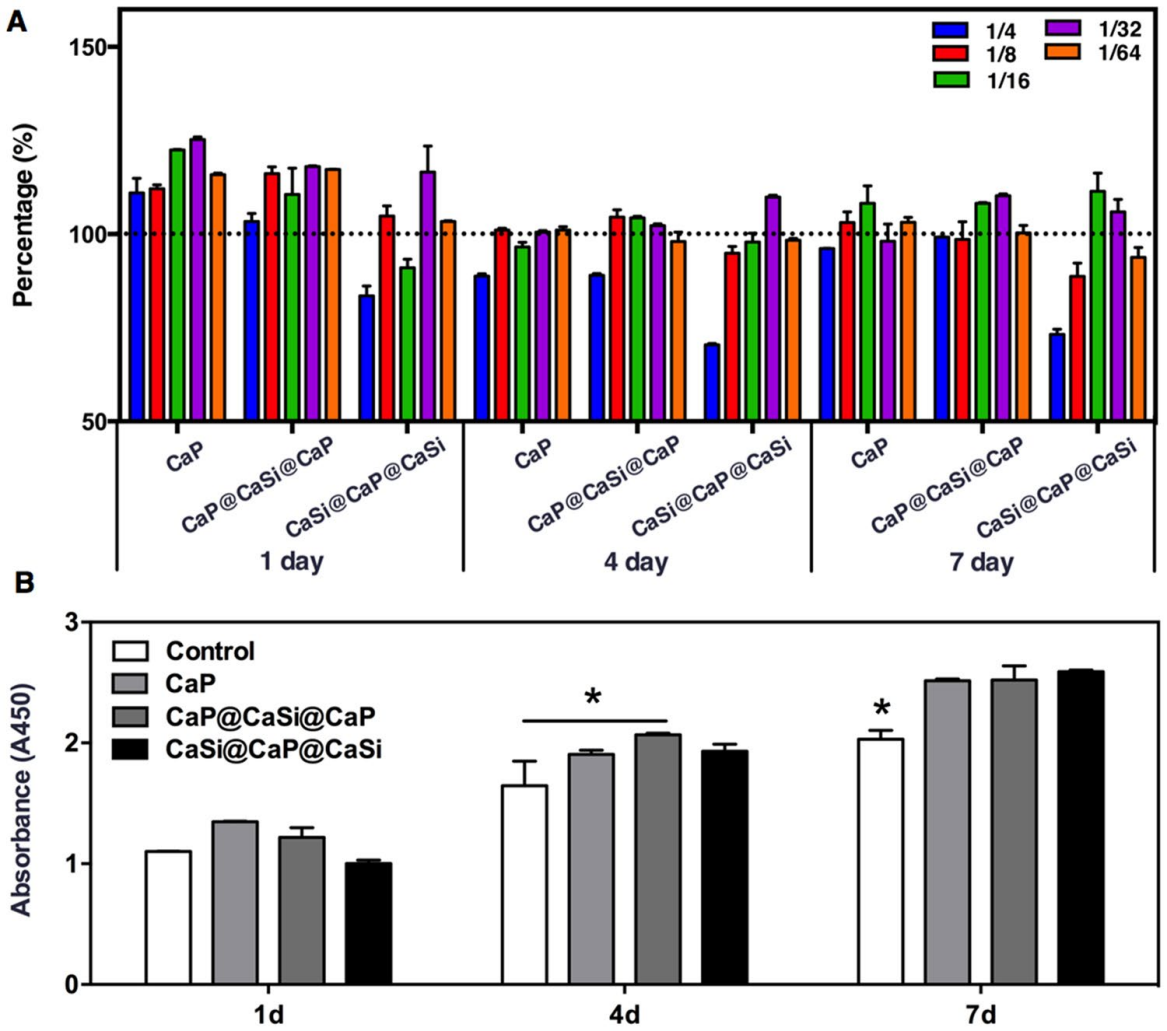
Cell proliferation of rBMSCs cultured in the presences of various concentrations of CaP, CaP@CaSi@CaP and CaSi@CaP@CaSi extracts after 1, 4, and 7 d culture (**A**) and in the optimal concentration (1/16 of 200 mg/ml) extracts of CaP, CaP@CaSi@CaP and CaSi@CaP@CaSi (B). *p < 0.05.

The CCK8 assay was performed to compare proliferation of rBMSCs (rat bone marrow- derived mesenchymal stem cells) in the diluted ionic extracts of microspheres (Fig. 2). Different dilution of extracts showed different impact on the proliferation of rBMSCs. Among the three groups of extracts, the CaSi@CaP@CaSi caused the earliest and highest suppressive effect on cell proliferation at high concentration of the extracts (1/4) while CaP showed the lowest (Fig. 2A). After 7 days of seeding, only at the 1/16 dilution that the proliferation of rBMSCs cultured in the extracts of all three groups showed a higher OD value than did rBMSCs cultured without any microspheres extracts (Fig. 2B). Therefore, the 1/16 dilution of the extract with similar higher proliferation was chosen as the optimal concentration for the following studies that required longer cultivation time up to 14 days.

### ALP activity of rBMSC exposed in conditioned media

**Figure 3:**
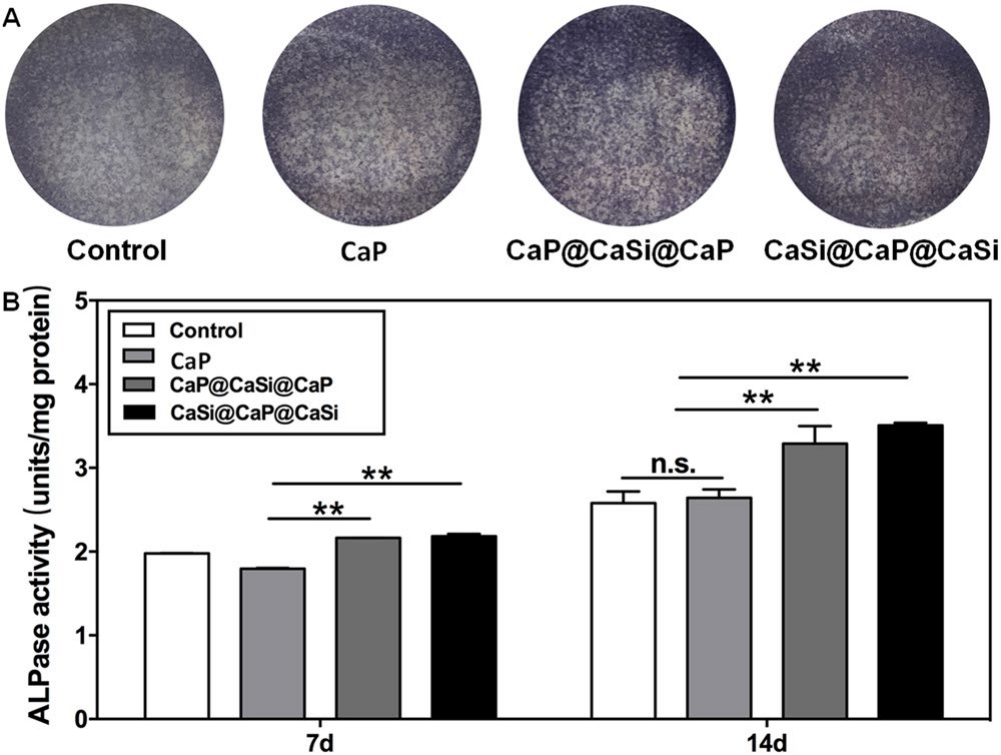
The ALP staining after 7 d of culture (**A**) and the activity analysis of rBMSCs cultured in medium without and with the ion extracts of CaP, CaP@CaSi@CaP, and CaSi@CaP@CaSi with optimal concentration (1/16 dilution) for 7 and 14 d of culture. **p < 0.01.

The ALP activity of rBMSCs cultured in the ionic extracts at the dilution of 1/16, and medium alone was examined. The ALP staining displayed that dual-shell microsphere extracts showed more intensive dying than that for CaP extract and medium alone at 7 d (Fig. 3A). The quantitative analysis showed that ALP activity increased over time. The CaSi@CaP@CaSi group and CaP@CaSi@CaP group induced higher ALP activity than did CaP group and the control after 14 days of culture (*p* < 0.05; Fig. 3B).

### Alizarin Red staining and quantitative analysis

**Figure 4:**
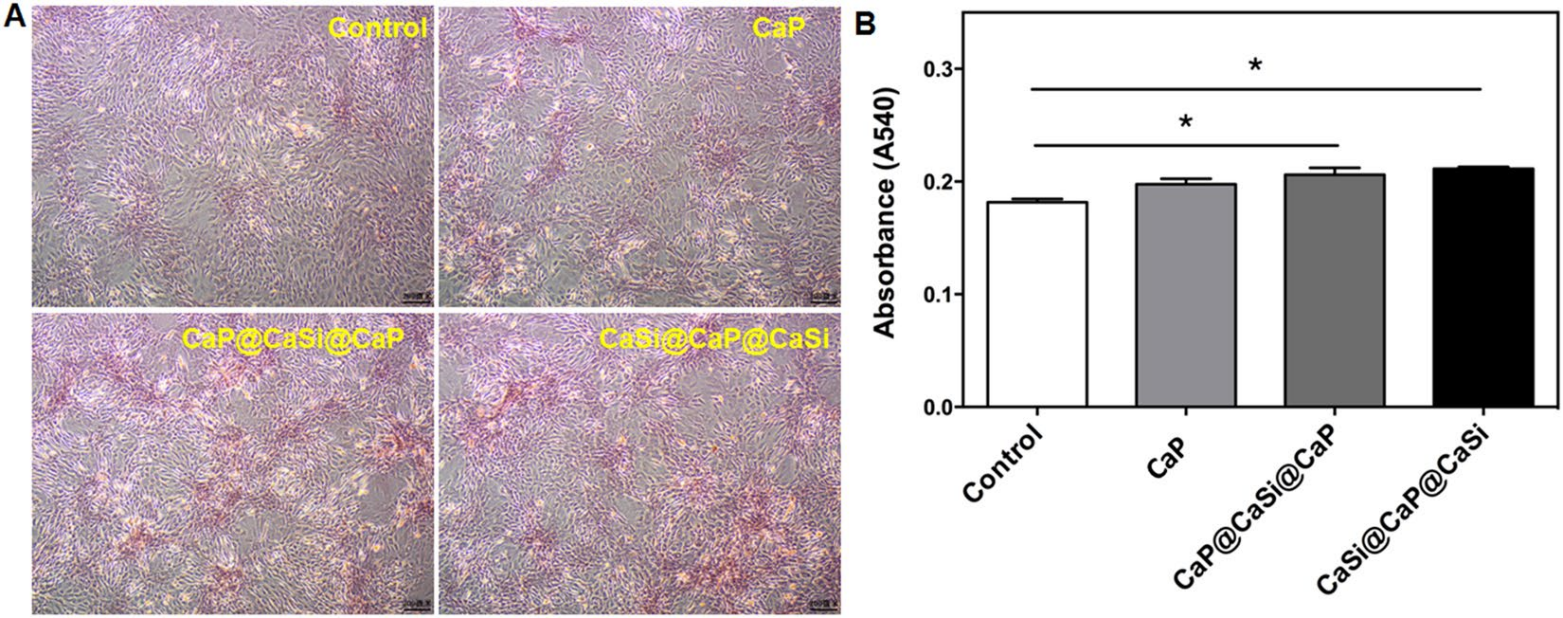
Alizarin Red staining and quantitative analysis of rBMSCs treated without and with CaP, CaP@CaSi@CaP and CaSi@CaP@CaSi extracts with optimal concentration (1/16 dilution) after 14 d (**A**) and quantitative analysis of the alizarin red staining. *p < 0.05.

The Alizarin Red staining was performed to show the nodule formation and calcium deposition. The color area and intensity of dual-shell microsphere groups were obviously larger and stronger than that of the CaP (Fig. 4A). Quantitative analysis of the alizarin red staining showed optical density of dual-shell microsphere groups were significantly higher than that in the control group (*p* < 0.05), but there were no significant differences between the dual-shell microspheres (*p* > 0.05).

### μCT analysis

**Figure 5:**
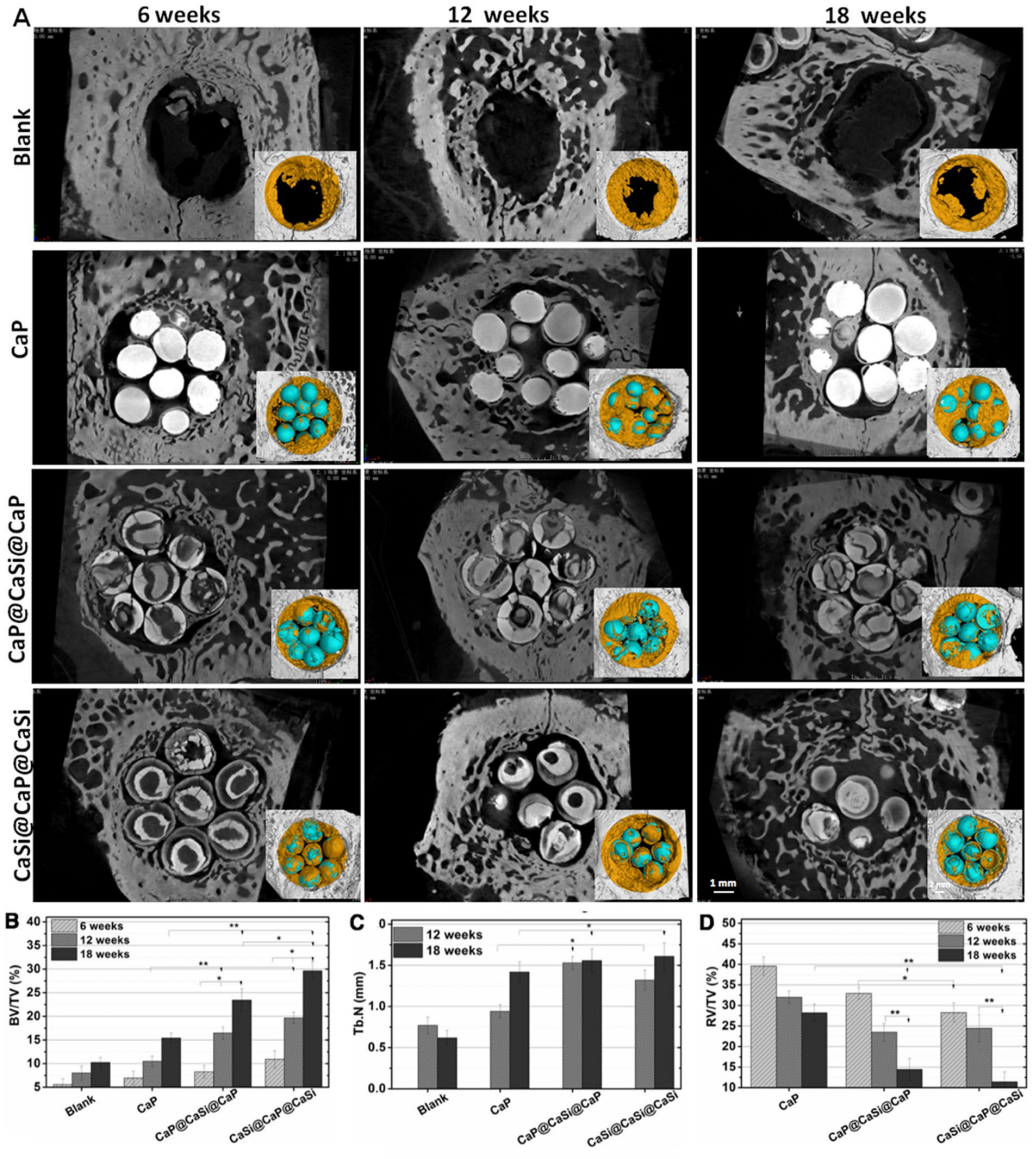
Representative 2D, 3D μCT images (**A**) and Quantitative parameters including BV/TV(**B**), Tb.N (**C**) and RV/TV (**D**) of the bone defects by μCT reconstruction software in the rabbit calvarial bone defects filled without and with CaP, CaP@CaSi@CaP, and CaSi@CaP@CaSi at 6, 12 and 18 week postoperatively (*n* = 5). The images inserted in (A) presenting the 3D μCT images of the calvarial bone defects filled with the bioceramic microspheres (blue) and newly formed bone tissue (yellow). *p < 0.05 and **p < 0.01.

Figure 5A showed the 2D, 3D μCT-reconstructed images of the bone defect. It could be seen that the critical-sized bone defect without filling any material (Blank group) remained non-healing cavity at 18 weeks postoperatively. The CaP microspheres were displayed as uniform high density images for the whole repair stage (18 weeks) accompanying with limited biodegradation. However, the dual-shell microspheres were displayed with high density and low density images alternatively, and the microspheres were degraded with time. It was showed that the CaSi phase was preferentially biodegraded either in the internal shell layer in CaP@CaSi@CaP microspheres or in the core and external shell layer in CaSi@CaP@CaSi microspheres. On the other hand, all the new bone started extending from the peripheral host bone at 6 weeks. Newly formed bone also scrambled onto the surface of the filled microspheres, except the Blank group. At 12 and 18 weeks, the amount of new bone increased and continued to gather towards the center from the outer ring of the defect. New bone tissue was infiltrated into the intervals among the microspheres and to connect with each other.

According to the quantitative analyses for the BV/TV, Tb.N and RV/TV based on the 3D μCT scan (Fig. 5B‒D), it was clear that the amount of newly formed bone increased in all microspheres groups from 6 to 18 weeks postoperatively. At 6 weeks, the BV/TV and Tb.N observed in all the groups showed no significant difference (*p* > 0.05). At 12 and 18 weeks, the BV/TV and Tb.N were both the highest in CaSi@CaP@CaSi group and the CaP@CaSi@CaP group was the second highest one, while the BV/TV in pure CaP group increased slowly. The RV/TV for the defects filled with the CaP (>25%) was significantly higher than other groups at 18 weeks (*p* < 0.01) and that of the CaSi@CaP@CaSi group was significantly lower than the CaP@CaSi@CaP group (*p* < 0.05) at both 12 and 18 weeks after surgery.

### Histological observation

**Figure 6:**
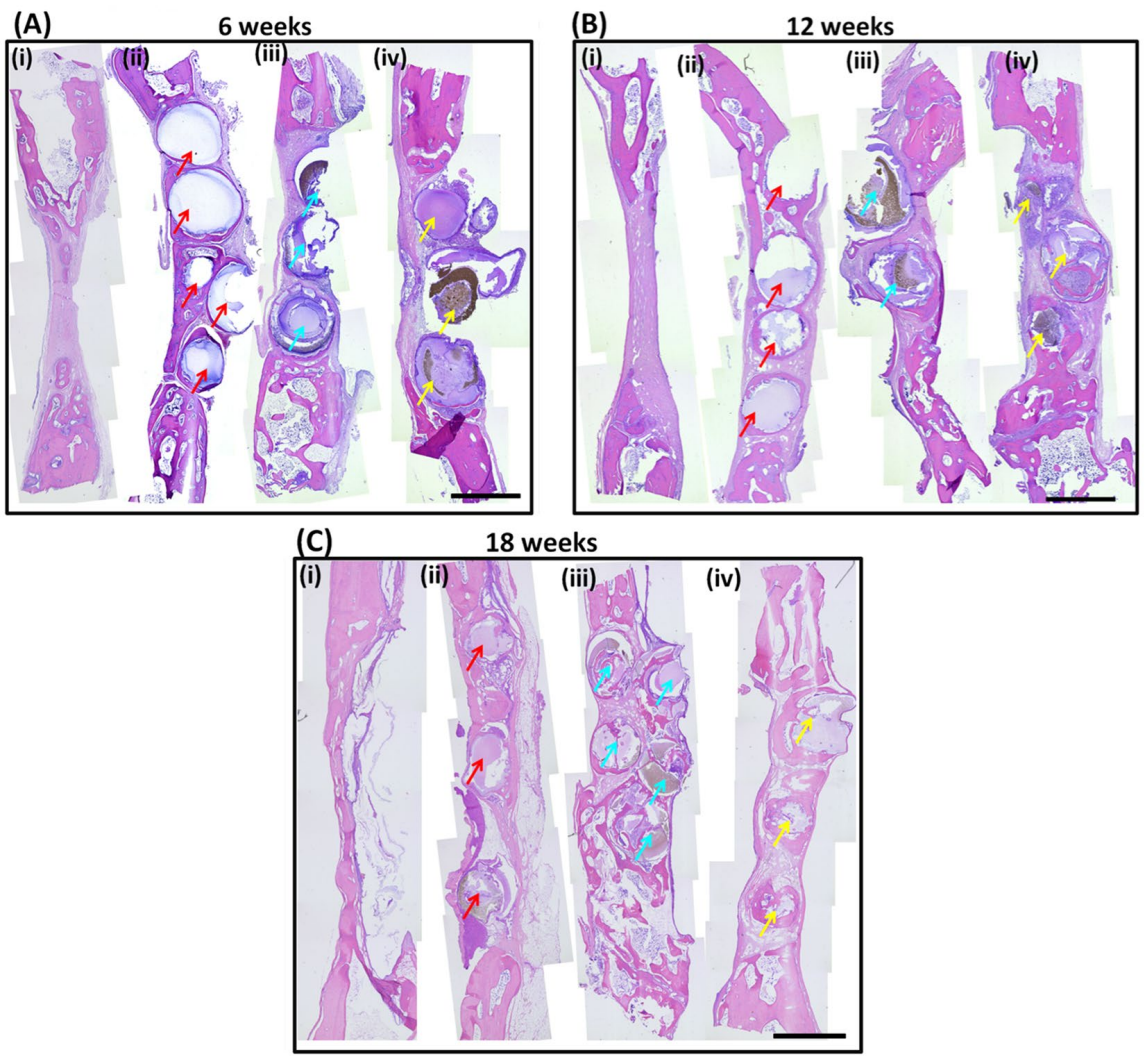
Macroscopically histological morphologies (HE staining of longitudinal section) in rabbit skull defects after implantation for 6–18 weeks. (i) Blank; (ii) CaP; (iii) CaP@CaSi@CaP; (iv) CaSi@CaP@CaSi. Original magnification 40×; Scale bar: 2 mm. Arrow, microspheres.

**Figure 7:**
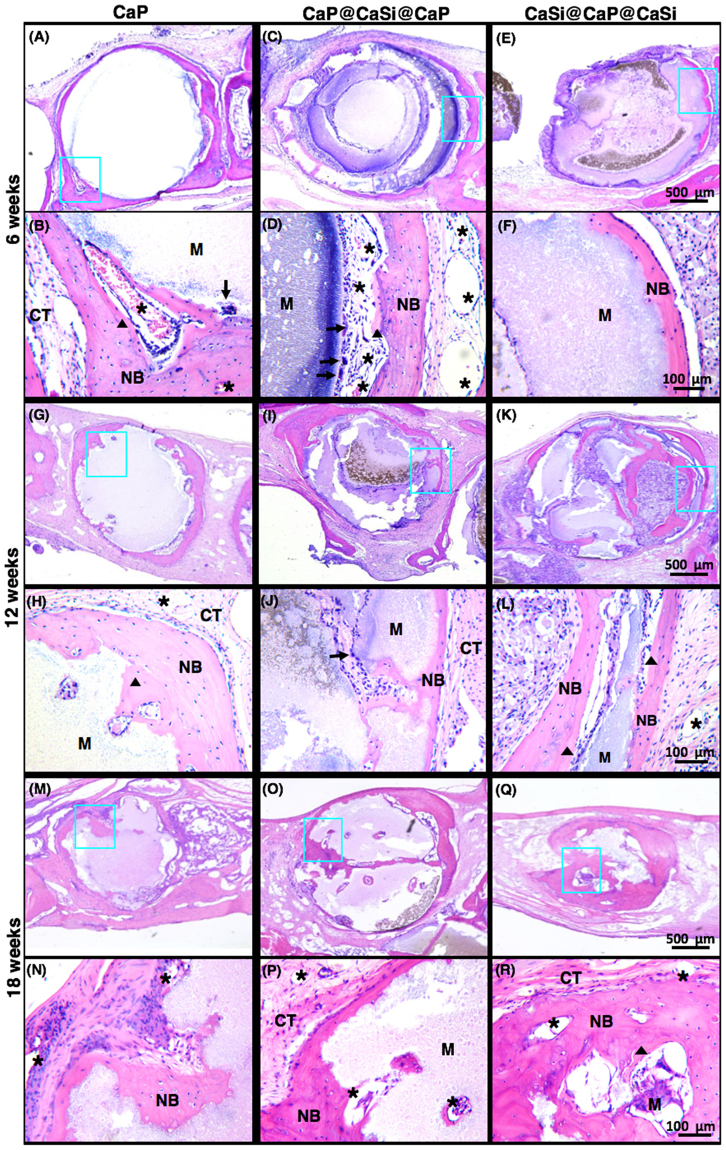
Low- and high-magnified histological morphologies (longitudinal section; HE staining) in rabbit skull defects after implantation for 6, 12 and 18weeks, respectively. Original magnification 40 and 200. CT, connective tissue; NB, new bone; M, materials; triangle, osteoid; star, blood vessel; triangle arrow, multinucleate cell.

Figures 6 and 7 showed the HE-staining histological images of the bone specimens. At 6 weeks after surgery, no obvious inflammation was observed in all the groups (Figs 6 and 7). In the pure CaP group, numerous multinucleate cells appeared around microsphere surface, and material was resorbed very slowly at 12 weeks. The newly formed bone was mainly present in the peripheral region of implants at 12 and 18 weeks (Figs 6Bii,Cii and 7G,H,M,N, respectively). In contrast, the degradation of dual-shell microspheres was higher and a thin layer of newly formed bone was observed around the surface of all microspheres. Multinucleate cells were observed directly onto the surface of the CaP@CaSi@CaP microspheres, while not found on the outer surface of CaSi@CaP@CaSi at 6 weeks. (Fig. 7C‒F). More vessels were also found near the surface of CaP and CaP@CaSi@CaP than that of CaSi@CaP@CaSi group at 6 weeks. As time went further to 18 weeks, vessels were observed in all the groups without obvious difference. New bone tissues invaded into most of the microspheres from the edge to the inner core as shown in rectangular frame in Fig. 7, and active osteoid tissues (labelled with triangle) were lined adjacent to the new bone (NB) at 12 and 18 weeks (Figs 6Biii,Biv,Ciii,Civ and 7I,K,O,Q, respectively). However, very limited amount of the pure CaP materials degraded and less newly formed bone tissue exhibited (Fig. 7M,N). Furthermore, less residual materials and more mature lamellar bone were observed in the CaSi@CaP@CaSi group compared with the CaP@CaSi@CaP group after 18 weeks (Figs 6Ciii, Civ and 7O,P,Q,R, respectively).

### Trap staining

**Figure 8:**
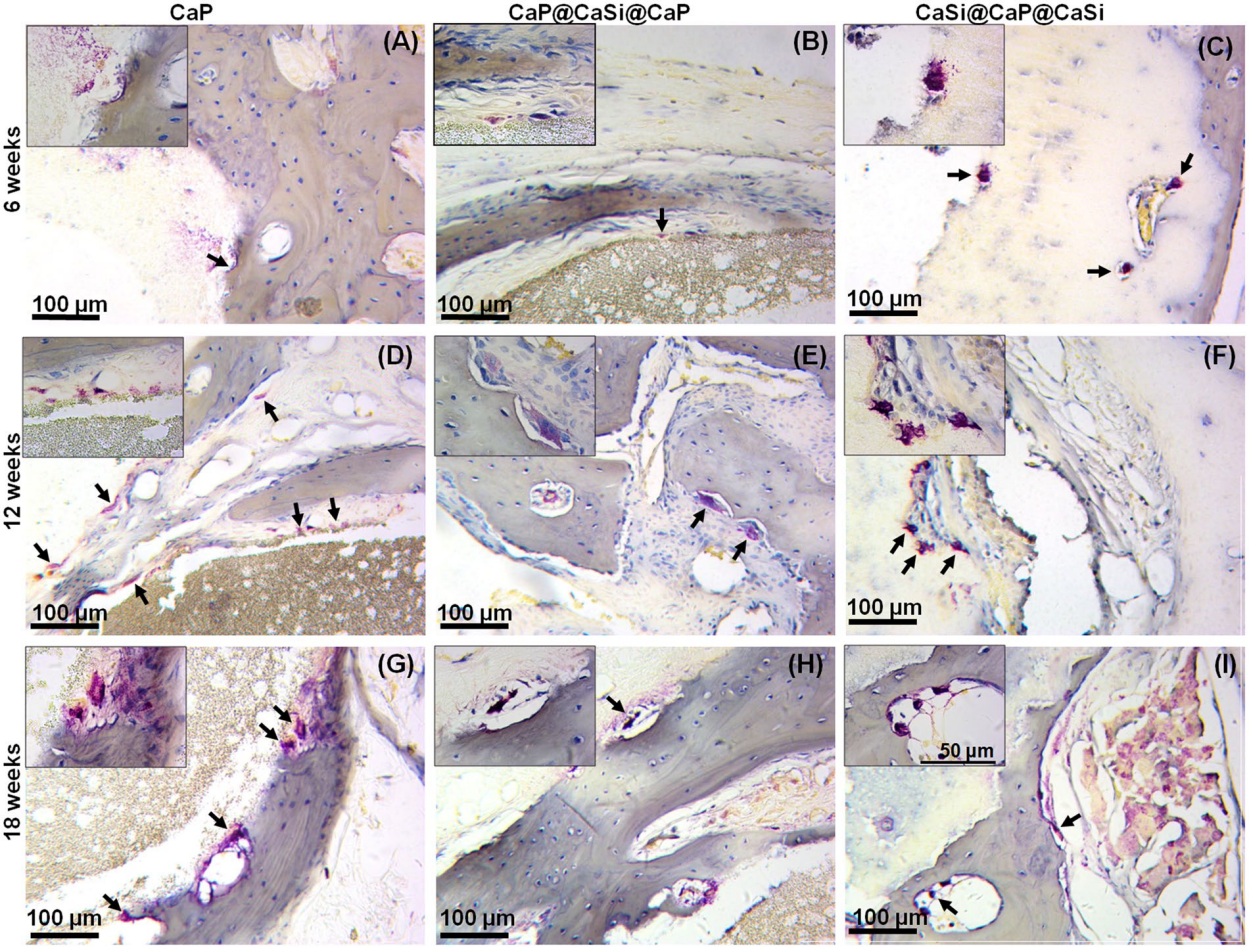
TRAP staining of CaP (**A**,**D**,**G**), CaP@CaSi@CaP (**B**,**E**,**H**), CaSi@CaP@CaSi (**C**,**F**,**I**) after implantation for 6, 12 and 18weeks, respectively. Insets representing higher magnification (400×). Arrow: TRAP-positive cells.

Osteoclastic activity was evaluated by tartrate-resistant acid phosphatase staining. At 6 weeks, the TRAP-positive cells were observed in the interface between material and new bone tissue in CaP and CaP@CaSi@CaP groups (Fig. 8A,B), while in the interior part of CaSi@CaP@CaSi group (Fig. 8C). At 12 and 18 weeks, TRAP-positive cells were observed on the surface of the materials and on the newly formed bone in all the groups (Fig. 8D–I). However, the morphology of the cells and the staining intensity in the cytoplasm varied. Slender TRAP-positive cells were observed in CaP group (Fig. 8D). Large osteoclast-like cells were found on the surface of newly formed bone with resorption lacunae in CaP@CaSi@CaP group (Fig. 8E), but the TRAP-positive cells were deeply stained and roundly shaped in CaSi@CaP@CaSi group (Fig. 8C,F).

## Discussion

Ideal bone substitutes are expected to have both osteoconductive and osteostimulative properties, and show a matched degradation rate with the new bone formation in a long term^25,26^. Bioceramic microspheres (~>500 μm in diameter) have been developed to treat bone defects, and these granules can be implanted into various shapes for ease of use^27^. The interconnected pores in the microsphere system can benefit drug delivery, osteoblastic cell migration and new bone tissue ingrowth^28,29^. However, the macropore enlargement in sparingly dissolvable diopside and HA microsphere system is very slow, which inevitably affect new bone tissue ingrowth^28,30^. Thus, we here to demonstrate an innovative approach to combine low degradable β-TCP with high degradable β-CaSi through double-shell hierarchy structure distribution instead of homogeneous hybrid.

CaP and CaSi could be easily integrated into the dual-shell microspheres which produced different biodegradation rates with time. The variation in biodegradation derived from the composition distribution in specific layer contributed to the bioactive ion release and surface bioactivity, and thus produced tunable osteostimulation in the calvarial bone defects. In fact, some previous studies have confirmed that the biodegradation rate of CaSi porous scaffolds and granules was overhigh for disadvantageous for mature bone formation in the bone defects^17,18^. Taking the thickness of thin-wall bone tissue in skull defect into account, the pure CaSi microspheres group was not included in this study.

The previous *in vitro* studies have demonstrated that the degradation rate of β-CaSi ceramics was apparently higher than that of β-TCP^20,31^. This is mainly because the solubility product constant of β-CaSi (2.5 × 10^−8^) is much higher than that of β-TCP (2.0 × 10^−29^), which suggested that faster dissolution of β-CaSi ceramics was mainly determined by the chemical composition of the materials. However, *in vivo* degradation studies of the β-CaSi porous scaffolds have showed that it is quickly biodegraded before new bone tissue remodeling which resulted in a non-healing osteogenesis^7^. Also, previous studies showed that high ion dissolution products from bioceramics could deteriorate cell viability^19^.

In this *in vitro* study, it is found that the 1/16 extract (200 mg/ml) of all bioceramic microspheres is the optimal one for cell proliferation, but the 1/4 extract of the CaSi@CaP@CaSi microspheres showed inhibitory effect. This is possibly attributed to the over-high Si concentrations in the conditioned cell culture medium. According to the internal diameters of the tri-nozzle system, the volume fraction of the dual-shell microspheres from inner core to external shell was 6.4%, 27.9%, and 65.7%, respectively. Theoretically, the mass fraction of CaP and CaSi in CaP@CaSi@CaP and CaSi@CaP@CaSi microspheres could be calculated as 73.99%/26.01% and 29.86%/70.14%, respectively. Thus, the CaSi phase in the external shell of CaSi@CaP@CaSi could readily dissolve in the cell culture medium. In contrast, the silicon release from internal CaSi shell of CaP@CaSi@CaP would be retarded by the external CaP shell though the CaP shell which demonstrated to be porous structure^32^.

Usually, ALP is expressed for the extracellular-matrix maturation that has been widely be used as a marker for osteoblast differentiation^33^, and Alizarin Red staining predicts the late mineralization phase of mature osteoblasts^34^. The current study indicates that both CaP@CaSi@CaP and CaSi@CaSi@CaP conditioned cell culture medium (1/16 dilution) is more beneficial for stimulating ALP expression and calcium nodule mineralization of BMSCs, as compared to pure CaP and the control. This may be contributed to the corporative effects of Ca ions and Si ions. Ca has been proven a potent effector on MSC differentiation, proliferation and differentiation^35^. An elevation in Ca could enhance osteoblastic differentiation^36^. The importance of Si in stimulating cell osteogenic differentiation *in vitro* has also been confirmed by previous studies^37,38^. The underlying mechanism through the activating ERK signaling pathway has been explained by Wang *et al*.^39^ and Zhang *et al*.^40^. While Han *et al*.^41^ suggested the WNT and SHH signaling pathway were also involved of the effect of silicate ions on proliferation and osteogenic differentiation of BMSCs. Thus, it may enhance the osteogenic cell proliferation, mineralization, and bone-related gene expression^39–41^.

Our evaluation *in vivo* showed that the value of BV/TV and Tb.N of CaP@CaSi@CaP group and CaSi@CaP@CaSi group measured by μCT quantitative analysis was remarkably higher than that of pure CaP group after 12 weeks of implantation. In the early stage at 6 weeks, no significant difference among defects filled with three groups of microspheres and unfilled was observed. The spontaneous healing capacity of surgically produced rabbit cranial defects might be taken into account^42^. Although spherical structure was designed for higher contact surface area and suitable shape fit for any irregular defect, the innate healing capacity originates from the defect margin might be impeded by the stuffed material which had a broad contact area with defect edges in the early stages^43^. Nevertheless, in the later stage, microspheres guided bone tissue stretching onto the surface and supported substantial bone regeneration while the unfilled group tended to collapse (Fig. 6A–C). On further increasing implantation to 12‒18 weeks, the BV/TV values were significantly higher for CaP@CaSi@CaP (16.47% and 23.43%) and CaSi@CaP@CaSi (19.66% and 29.65%) than the CaP group (10.5% and 15.43%) at 12 and 18 weeks (Fig. 5). Meanwhile, the CaP group degraded significantly slower than the groups containing β-CaSi. On one hand, the prolonged existence of residual materials might limit the space for bone and vascular tissues to grow^44,45^. On the other hand, this superiority of bone regeneration might be ascribed to the chemical composition of the materials in the presence of CaSi. For CaP@CaSi@CaP group, the porous nature of CaP shell enabled the release of bioactive Si ions through the eternal shell layer. As the external shell layer of CaSi@CaP@CaSi, the rapid biodegradation of CaSi layer may induce an increase in silicon ion concentration *in situ*. Silicon is found to be essential for normal bone growth and development^46^. Wang *et al*. found that introduction of 50% and 80% wt% β-CaSi into β-TCP could dramatically enhance the amount of newly formed bone in the long term up to 26 weeks^17^. Similar results were observed previously^21,24^. However, our results were superior to these studies that the new bone tissue could not only migrate into the macropore of the microsphere scaffolds, but also readily invade into the dual-shell microspheres from the edge to the inner core with the preferential biodegradation of CaSi phase *in vivo* (Fig. 7I,J,Q,R).

Another interesting aspect for the present study is to compare the osteogenic efficiency of the composition distribution of CaP and CaSi in dual-shell microspheres. The μCT analysis showed that there is no significant difference in the value of Tb.N at 12 and 18 weeks (Fig. 5C), whereas the BV/TV and RV/TV data between the dual-shell microsphere groups is significantly different at 18 weeks. The CaSi@CaP@CaSi group displayed a higher value of BV/TV than the other group (29.8% & 23.5%; Fig. 5B). The quantitative analysis revealed a distinct difference in RV/TV between CaP@CaSi@CaP and CaSi@CaP@CaSi in material biodegradation (Fig. 5D). From 12 to 18 weeks, the RV/TV in CaSi@CaP@CaSi group was decreased remarkably, but a mild decrease was determined for the CaP@CaSi@CaP group. On the other hand, the newly formed bone in CaSi@CaP@CaSi group was not only around the microspheres but also in the inner core layer at 12 and 18 weeks after implantation (Fig. 6Biv,Civ). The central bone was connected to the periphery bone or existed in a form of bony islet separated from the defect margin, which we considered to have been stimulated by the Si ions released from the core layer of CaSi. In contrast, the newly formed bone in the CaP@CaSi@CaP group mainly existed encircling the entire body. This phenomenon may be associated with the biochemical properties of CaP and β-CaSi, and their specific locations in the microspheres. From the chemical point of view, the solubility of β-CaSi is much higher than that of β-TCP in physiological environment which indicates a likely faster bio-dissolution of β-CaSi *in vivo*^7^. As for the closely packed bioceramic microsphere system, the primary porosity is very low (~30%), and the interconnected pore size is directly associated with the diameter of microspheres. Along with the biodegradation of microspheres, the interconnected pore size increases facilitating new tissue ingrowth. Therefore, in CaP@CaSi@CaP group, the slow degradation of CaP in core layer and external shell layer *in vivo* may allow a comparative slow new bone tissue ingrowth. And in CaSi@CaP@CaSi group, it showed an appreciable bioresorption at the early stage and for a long-term stage (Figs 5, 6 and 7), thus a clear spatiotemporal evolution from material to new bone tissue took place in the gap of microspheres. The low-biodegradation CaP internal retained for supporting new bone remodeling.

**Figure 9:**
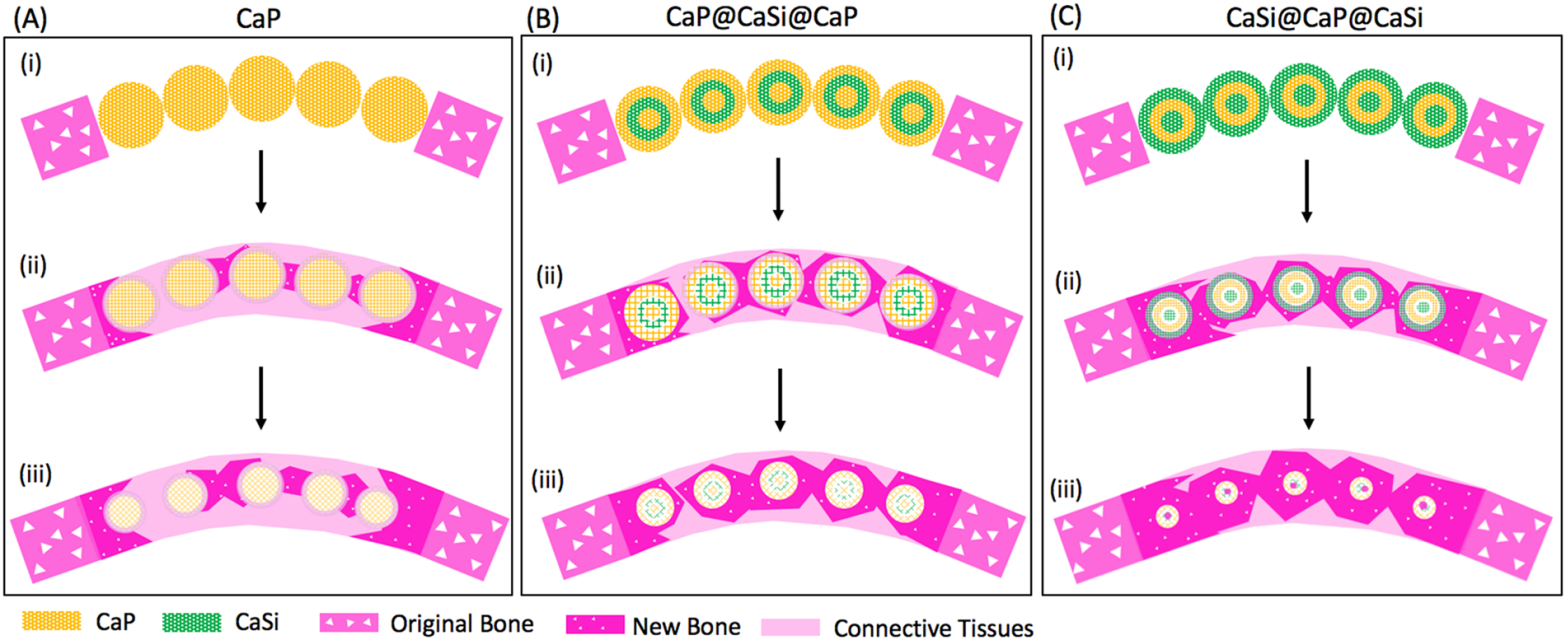
A schematic diagram for biodegradation and matched bone regeneration of packed microspheres with time in calvarial bone defect. (**A**) CaP; (**B**) CaP@CaSi@CaP; (**C**) CaSi@CaP@CaSi.

To summarize, a schematic diagram could be used to illustrate the different new bone regeneration pattern due to the different interconnected pore evolution of the three groups of bioceramic microspheres in calvarial defects (Fig. 9). In fact, all bioceramic microsphere arrays were initially closely packed in the calvarial bone defects (Fig. 9Ai,Bi,Ci). Then, the external surface exhibited different biodegradation rate, which resulted in different enlargement of the macroporous structures between microspheres, and meanwhile the internal microporous structures in different component layers were varied differently. The biodegradation of biphasic bioceramic microspheres and bioactive ion release would readily produce large enough interconnected pore architectures for cells migration and nutrient infiltration. Based on these different composition distribution and biodegradation characteristics, it is reasonable to assume that the pure CaP microspheres underwent a very slow biodegradation with time; but as for the CaP@CaSi@CaP and CaSi@CaP@CaSi microspheres, the biodissolution of CaSi layer in the external or internal shell layer and the calcium and silicon release through the porous CaP external shell layer is thought to be favorable promoting more new bone regeneration and ingrowth. With the prolongation of implantation time, the external CaSi shell layer of CaSi@CaP@CaSi microspheres were degraded completely and the sparingly dissolvable internal CaP shell would support the new bone remodeling and maturation (Fig. 9Ciii). The core CaSi part gradually biodegraded and was replaced by new bone tissues; but in contrast, the new bone tissue ingrowth in the CaP@CaSi@CaP group would be retarded due to the slow-biodegradation nature of the external CaP shell layer.

The TRAP staining results showed that TRAP-positive cells appeared on the interface between all the microspheres and new bone tissue at 6‒18 weeks, which means cell-mediated resorption is involved. Besides, the material was gradually resorbed and replaced by new bone tissues. TRAP-positive multinucleate cells were observed on both the surface of β-TCP and β-CaSi in other studies^7,47,48^. Since the type 5 isoenzyme of acid phosphatase is a lysosomal enzyme found in osteoclasts, TRAP staining in this study was to show the activity of osteoclasts^49^. At 6 weeks, in the CaP and CaP@CaSi@CaP groups the TRAP-positive cells and multinucleate cells were observed in the interface between material and newly formed bone in Trap-staining and HE staining, respectively (Figs 7B,D and 8A,B). It suggests that cell-mediated CaP degradation is involved. It is also observed previously that the degradation of CaP bioceramics can occur by both solution- mediated dissolution and activities of osteoclastic cells^50^. Nevertheless, for CaSi@CaP@CaSi group, neither multinucleate cells in HE staining nor TRAP-positive cell were found in the junctional zone between the external shell layer and the newly formed bone (Figs 7F and 8C). The TRAP-positive cells were observed interior of CaSi@CaP@CaSi where were more likely to be CaP layer. These results may indicate a mainly fast dissolution-mediated degradation of the CaSi shell of CaSi@CaP@CaSi. Other study also observed multinucleate cells were hardly observed on the rapidly degraded particles *in vivo*^20^. Studies *in vitro* demonstrated that soluble Si could inhibit osteoclast phenotypic genes expressions, osteoclast formation and bone resorption^50,51^. However, the role of osteoclasts-involved biodegradation of CaSi is still controversial as Xu *et al*. have observed trap-positive multinucleate cells on the surface of CaSi^7^. At 12 and 18 weeks, respectively, TRAP-positive cells were observed active on both interface of all the microspheres and newly formed bone, implying remodeling of the new bone tissue.

## Conclusions

The *in vitro* cell responses and *in vivo* osteogenic efficacy of the dual-shell CaSi/CaP microspheres were comprehensively assessed. It is demonstrated that their appropriately diluted ion extracts may enhance ALP activity and nodule formation, indicating that their potent ability for mediating osteogenic differentiation of rBMSCs. When implanted in calvarial defects, the dual-shell microspheres displayed appreciable bone ingrowth when compared to β-TCP. The specific distribution of CaP and CaSi in dual-shell structure resulted in different regeneration patterns with time. The single layer of CaSi@CaP@CaSi microsphere arrays in such thin-wall bone defects showed a superior performance in balancing the biodegradation and bone regeneration through full utilization of the physicochemical and biological properties of β-TCP and β-CaSi. These results suggest that the gradient distribution in such dual-shell microspheres with different biodegradable components readily tailor the biodegradation of core or internal shell layer and avoid the structural collapse of scaffolds, that is helpful for exerting respective effect comparative independently.

## Materials and Methods

### Preparation and characterization of dual-shell microspheres

The β-TCP and β-CaSi powders were prepared by the conventional chemical precipitation method.^24^ To obtain dual-shell microspheres, β-TCP and β-CaSi powders were added into sodium alginate hydrogel (15 wt%) with constant stirring, respectively. The two slurries went through the co-concentric tri-nozzle (diameter: Ø2.0 mm, Ø1.4 mm, Ø0.8 mm) through different micro-tubes, resulting in dual-shell droplets, and then the granules were collected by 0.5 M Ca(NO_3_)_2_ solutions. Depositing different components in the core or shell layers, two kinds of dual-shell CaP@CaSi @CaP and CaSi@CaP@CaSi granules were created. The microspheres were washed by deionized water three times. After drying at 80 °C, the particles were finally sintered at 1150 °C for 3 h. The pure CaP microspheres were prepared by extruding the β-TCP slurry while the other condition remained the same. The fracture surface and chemical analysis of the microspheres was characterized using scanning electric microscopy (SEM, SIRION-100, FEI, USA) with energy-dispersive spectroscopy (EDX).

### Preparation of biomaterial extracts

The ionic extracts of pure CaP and dual-shell microspheres were prepared in accordance with the International Organization for Standardization (ISO 10993-12). The original solution of 200 mg/ml was prepared by immersing 1.0 g of microspheres into 5 ml serum-free Dulbecco’s Modified Eagle’s Medium (DMEM, Gibco) and incubated at 37 °C for 24 h. Then the supernatant was collected, centrifuged and sterilized through a 0.22-μm Millipore membrane as original extract for further use. The Ca, P, and Si concentrations in the extracts were measured by using inductively coupled plasma optical emission spectroscopy (ICP-OES: 710-ES, Varian, USA).

### Cell proliferation assay

All the animal procedures including *in vivo* animal study were performed in accordance with the ARRIVE guidelines^52^ and regulations of laboratory animal use of Zhejiang University (no.866, Yuhangtang Road, Hangzhou, P.R. China). The study protocol was reviewed and approved by the Animal Care and Experiment Committee of Zhejiang University (ZJU20160455). Rat bone marrow-derived mesenchymal stem cells (rBMSCs) were obtained from the femora of 3‒4-week old Sprague- Dawley rats. To determine the proper concentration of the ionic extracts for following study, a serial of diluted extracts with 1/4, 1/8, 1/16, 1/32, and 1/64 concentrations were prepared by diluting the original extracts with serum-free DMEM. The BMSCs were seeded in 96-well plates at 5 × 10^3^ cells/well. After 24 h, the culture medium was replaced by various concentrations of the microsphere extracts supplemented with 10% fetal bovine serum (Gibco) and then cultured for 1, 4 and 7 d, respectively. The cell viability was evaluated using the Cell Counting Kit-8 (CCK-8; Dojindo Molecular Technologies, Tokyo, Japan) according to the manufacturer’s instructions. At each time point, the culture medium was changed with CCK-8 mixture solution containing 10% volume of CCK-8 solution. After incubation for 2 h at 37 °C, 100 μl of the reaction solution was transferred to a new 96-well plate, and the optical density was measured at 450 nm using a multifunctional microplate reader (SpectraMax M5, Molecular Devices, USA). All experiments were performed in triplicate, and the results were shown as units of optical density (OD) absorbance value. And the results were shown as the ratio of optical density (OD) absorbance value of experimental groups divided by that of the control group.

### Alkaline phosphatase (ALP) staining and activity assay

To investigate the early differentiation of BMSCs stimulated by the ionic extracts, the BMSCs were seeded in 6-well plates at a density of 1 × 10^5^ cells/well and cultured in DMEM with 1/16 concentration of microsphere extracts described above. The cell layers at day 7 and 14 were rinsed with phosphate-buffered saline (PBS) and fixed with 4% paraformaldehyde for 15 min at 4 °C. The fixed cells were immersed in a mixture of BCIP/NBT working solution (Beyotime, Jiangsu, China) for 30 min at room temperature and then washed with PBS. The stained samples were then observed. The levels of ALP activity at day 7 and 14 were measured according to the manufacturer’s instruction (Wako, Japan). Briefly, the cell culture supernatant was collected and centrifuged at 15,000 rpm for 5 min at 4 °C. 20 μl of supernatant was added into 100 μl working assay solution. After incubation at 37 °C for 15 min, the reaction was stopped by adding 80 μl stop solution to each well. Immediately the absorbance at 405 nm was measured with microplate reader (SpectraMax M5, Molecular Devices, USA). Total protein content was determined of the same samples using BCA Protein Assay Kit (Takara). The relative ALP activity was expressed by normalizing the amount of nitrophenol released with total amount of cellular protein.

### Alizarin Red Staining and quantitative analysis

In order to identify the mineralization, Alizarin Red staining was performed on day 14 after the BMSCs were cultured in DMEM with 1/16 concentration of microsphere extracts described above. The cells were fixed in 4% paraformaldehyde for 15 min and then washed with deionized water, the photos were taken under light microscope (Zeiss AX10; Germany). For quantitative analysis, the staining was dissolved in 10% cetylpyridinuchlotide (Sigma) in 10 mM sodium phosphate (Aladdin) and the ODs were measured at 540 nm using a microplate reader (SpectraMax M5, Molecular Devices; USA).

### Implantation of microspheres in calvarial bone defects of rabbits

Before animal surgery, the microspheres were sterilized by autoclaving. Fifteen male New Zealand white rabbits weighting 2.8‒3.0 kg were used in this study. Under general anesthesia by intravenous injection of pentobarbital sodium (30 mg/kg, Sigma), rabbits were placed in prone position. After shaving and disinfection of the operation areas, a longitudinal incision was made along the midline of the scalp. Full-thickness skin was flapped and periosteum was bluntly dissected to expose the cranium surface. Four separated circular defects were created in the cranium using an 8-mm diameter trephine bur without damaging the duramater under 0.9% physiologic saline constant irrigation. The four circular defects were randomly filled with three groups of microspheres or left the implant-free defects as Control. Operative areas were stratified sutured, and then penicillin (400,000 U/d) was administered through intramuscular injection for 3 d. The rabbits were euthanized at 6, 12, 18 weeks (*n* = 5) after surgery. The cranium containing the materials were harvested and fixed for 48 h in 4% paraformaldehyde for further study.

### Micro-computed tomographic analysis

Three-dimensional micro-computed tomography (μCT) (Y. Cheetah, Y. XLON, German) was used to assess bone formation in the defects, at a voltage of 80 kV, an electric current of 62.5 μA and a projection number of 720. Then 3D images were reconstructed using the software VGStudio Max 2.2. The amount of bone regeneration was evaluated by analyzing bone volume proportion of the total defect volume (BV/TV), trabecular number (Tb.N) and residual material volume proportion of the total defect volume (RV/TV).

### Histological analysis

Following μCT analysis, all the specimens were immersed in EDTA decalcifying solutions for 3 weeks with changing of fresh decalcifying solutions every 3 d. Then, specimens were dehydrated in graded series of alcohol solution and finally embedded in paraffin. A series of 5 μm thick sections were cut perpendicular to the horizontal plane of the circular defect and stained with hematoxylin and eosin (H&E).

#### TRAP staining

Serial sections were stained for tartrate-resistant acid phosphate (TRAP) with commercial TRAP kit (Sigma, St Louis, USA). The sections were incubated in the incubation solution made up of Fast Garnet GBC Base Solution, sodium nitrate solution, naphthol AS-BI phosphoric acid, acetate solution, tartrate solution and deionized water for 60 min at 37 °C.

### Statistical analysis

The data were expressed as mean ± standard deviation. Statistical analyses were carried out by a one-way analysis of variance (one-way ANOVA) and Turkey test for multiple comparison tests using GraphPad Prism 6.0. In all cases, a *p* < 0.05 was considered statistically significant.
